# *Alu* Recombination-Mediated Structural Deletions in the Chimpanzee Genome

**DOI:** 10.1371/journal.pgen.0030184

**Published:** 2007-10-19

**Authors:** Kyudong Han, Jungnam Lee, Thomas J Meyer, Jianxin Wang, Shurjo K Sen, Deepa Srikanta, Ping Liang, Mark A Batzer

**Affiliations:** 1 Department of Biological Sciences, Louisiana State University, Baton Rouge, Louisiana, United States of America; 2 Biological Computation and Visualization Center, Louisiana State University, Baton Rouge, Louisiana, United States of America; 3 Center for BioModular Multi-Scale Systems, Louisiana State University, Baton Rouge, Louisiana, United States of America; 4 Department of Cancer Genetics, Roswell Park Cancer Institute, New York, United States of America; Fred Hutchinson Cancer Research Center, United States of America

## Abstract

With more than 1.2 million copies, *Alu* elements are one of the most important sources of structural variation in primate genomes. Here, we compare the chimpanzee and human genomes to determine the extent of *Alu* recombination-mediated deletion (ARMD) in the chimpanzee genome since the divergence of the chimpanzee and human lineages (∼6 million y ago). Combining computational data analysis and experimental verification, we have identified 663 chimpanzee lineage-specific deletions (involving a total of ∼771 kb of genomic sequence) attributable to this process. The ARMD events essentially counteract the genomic expansion caused by chimpanzee-specific *Alu* inserts. The RefSeq databases indicate that 13 exons in six genes, annotated as either demonstrably or putatively functional in the human genome, and 299 intronic regions have been deleted through ARMDs in the chimpanzee lineage. Therefore, our data suggest that this process may contribute to the genomic and phenotypic diversity between chimpanzees and humans. In addition, we found four independent ARMD events at orthologous loci in the gorilla or orangutan genomes. This suggests that human orthologs of loci at which ARMD events have already occurred in other nonhuman primate genomes may be “at-risk” motifs for future deletions, which may subsequently contribute to human lineage-specific genetic rearrangements and disorders.

## Author Summary

The recent sequencing of a number of primate genomes shows that small segments of DNA known as *Alu* elements are found repeatedly along all chromosomes, and indeed comprise ∼10% of the human genome. Although older *Alu* elements that have been in the genome for a long time accumulate some random mutations, overall these elements retain high levels of sequence identity among themselves. The presence of many near-identical *Alu* elements located close to each other makes primate genomes prone to DNA recombination events that generate genomic deletions of varying sizes. Here, by scanning the chimpanzee genome for such deletions, we determined the role of the *Alu* recombination-mediated deletion process in creating structural differences between the chimpanzee and human genomes. Using a combination of computational and experimental techniques, we identified 663 deletions, involving the removal of ∼771 kb of genomic sequence. Interestingly, about half of these deletions were located within known or predicted genes, and in several cases, the deletions removed coding exons from chimpanzee genes as compared to their human counterparts. *Alu* recombination-mediated deletion shows signs of being a major sculptor of primate genomes and may be responsible for generating some of the genetic differences between humans and chimpanzees.

## Introduction

Mobile elements are a major source of genetic diversity in mammals [1,2]. *Alu* elements, a family of short interspersed elements (SINEs), emerged ∼65 million y ago (Mya) and have successfully proliferated in primate genomes with >1.2 million copies [2–5]. *Alu* elements consist of a left monomer and a right monomer [2,6]. Each of these monomers independently evolved from 7SL-RNA [7] and subsequently fused into the dimeric *Alu* element in the primate lineage [6]. *Alu* elements are known to be associated with primate-specific genomic alterations by several mechanisms, including de novo insertion, insertion-mediated deletion, and unequal recombination between *Alu* elements [8–11]. The *Alu* family consists of a number of subfamilies, which maintain high sequence identity among themselves (70%–99.7%) [12–15].

Mispairing between two *Alu* elements has been shown to be a frequent cause of deletion or duplication in the host genome [10,11,16]. A recent study of human-specific *Alu* recombination-mediated deletion (ARMD) reported a significant number of events associated with *Alu* elements [10]. An ARMD may arise through either interchromosomal recombination by mismatch of sister or nonsister chromatids during meiosis [17] or by intrachromosomal recombination between two *Alu* elements on the same chromosome. Previously, Sen et al. [10] found 492 human-specific ARMD events responsible for ∼400 kb of deleted genomic sequence in the human lineage [10]. Here, we report 663 chimpanzee-specific ARMD events identified from comparative analysis of the chimpanzee and human genomes. The chimpanzee-specific ARMD events deleted a total of ∼771 kb of genomic sequence in chimpanzees, including exonic deletions in six genes, sometime after the divergence of the human and chimpanzee lineages (∼6 Mya). ARMD events in the chimpanzee genome have generated large deletions (up to ∼32 kb) relative to human-specific ARMD events. Taking deletions in both the human and chimpanzee lineages into account, we suggest that ARMD events may have contributed to genomic and phenotypic diversity between humans and chimpanzees.

## Results

### A Genome-Wide Analysis of Chimpanzee-Specific ARMD Events

To investigate chimpanzee-specific ARMD loci, we first computationally compared the chimpanzee (panTro1) and human (hg17) genome reference sequences. A total of 1,538 ARMD candidates were initially retrieved using panTro1. These loci were converted to panTro2 (March 2006), which, due to the better quality of the sequence assembly, allowed us to eliminate a number of loci that mimicked authentic ARMD loci. Through a comparison of panTro1 and panTro2, we discarded 258 of the 1,538 loci (Table 1). The remaining 1,280 loci were manually inspected using the repetitive DNA annotation utility RepeatMasker (http://www.repeatmasker.org/cgi-bin/WEBRepeatMasker). In terms of local sequence architecture, human-specific mobile element insertions between two preexisting adjacent *Alu* elements could be computationally confused with a chimpanzee-specific deletion. Because the consensus sequences of the human-specific mobile elements (e.g., *Alu*Yb8, *Alu*Ya5, SVA, and L1Hs) have been well established in RepeatMasker, we were able to identify and eliminate from our analysis 189 human-specific insertion loci, including processed pseudogenes. The remaining 1,091 candidate ARMD loci were inspected using triple alignments of human (hg18), chimpanzee (panTro2), and rhesus macaque (rheMac2) sequences at each locus, and also on the basis of their target site duplication (TSD) structures (see Materials and Methods). After manual inspection, 342 of the candidate ARMD loci were examined by PCR to verify their status as authentic ARMD loci. Finally, combining computational and experimental results, 663 loci were confirmed as bona fide chimpanzee-specific ARMD loci (Table 1 and Dataset S1).

**Table 1:**
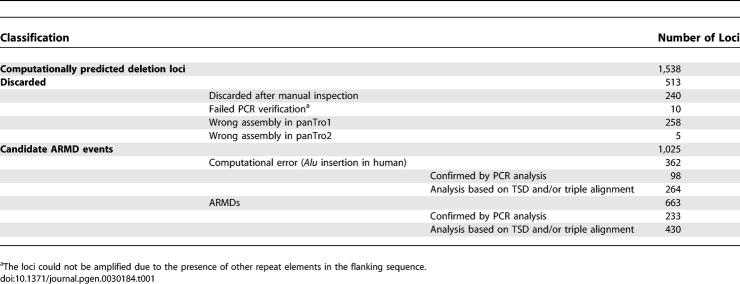
Summary of Chimpanzee-Specific ARMD Events

In this study, we combined computational data mining and wet-bench experimental verification, an approach that is optimal for identifying lineage-specific insertions and deletions [10]. Whereas Sen et al. [10] computationally compared the human and chimpanzee genomes, in our analysis, the draft version of the rhesus macaque genome sequence was used as an outgroup when filtering computational output for false positives (see Materials and Methods). This allowed us to eliminate 215 candidate ARMD loci prior to wet-bench verification, minimizing the cost and time needed to confirm authentic chimpanzee-specific ARMD events, as compared with the previous human-specific ARMD study.

### Genomic Deletion Through Chimpanzee-Specific ARMD Events

**Figure 1:**
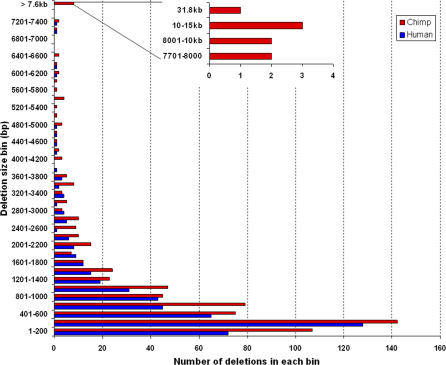
Size Distribution of Chimpanzee-Specific ARMD Events Size distribution of chimpanzee-specific ARMD events (red bars) compared with that of human-specific ARMD events (blue bars), displayed in 200-bp bin sizes.

Since the human-chimpanzee divergence ∼6 Mya, chimpanzee-specific ARMD events have occurred 1.3 times as often as their human-specific counterparts (663 chimpanzee-specific versus 492 human-specific events). The total amount of genomic DNA deleted by ARMD events from the chimpanzee genome is estimated to be 771,497 bp. However, when we consider that the average indel divergence between the human and chimpanzee genomes has been estimated at 5.07% [18], the precise amount of DNA deleted through ARMDs in the chimpanzee genome could be anywhere between ∼733 and ∼811 kb (±5.07% of ∼771 kb). The size distribution of DNA sequences deleted through chimpanzee-specific ARMD events ranged from 111 to 31,861 bp, with 1,164 bp average and 615 bp median ARMD sizes. Similar to the pattern observed in human-specific ARMD events [10], a histogram of the size distribution of chimpanzee-specific ARMDs is skewed toward deletions of shorter size, with ∼68% (449 of 663) of the deletion events shorter than 1 kb (Figure 1). As expected, about 70% of the deleted genomic DNA sequences are composed of repetitive elements (Table 2), of which *Alu* element sequences account for ∼64% (338 kb of 528 kb). Interestingly, the amount of sequence deleted through the ARMD process from the chimpanzee genome is twice as much as that from the human genome during the same period of time. Ten chimpanzee-specific ARMD events were found to have each deleted >7.3 kb of sequence (Figure 1); ARMD sizes this large were not observed in the human-specific study. Among these, the largest deleted sequence is 31,861 bp in length, within which only the *SLC9A3P2* pseudogene and two intergenic regions are found in the ancestral sequence (i.e., human ortholog).

**Table 2:**
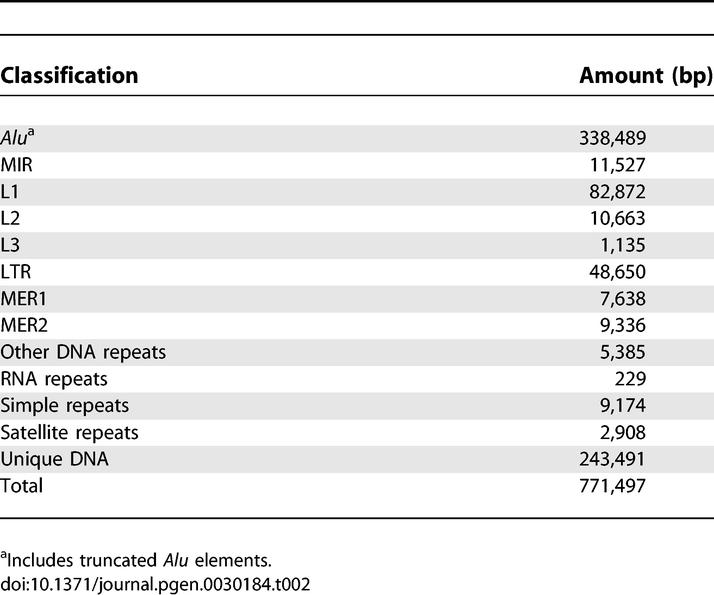
Classification of Genomic DNA Deleted by ARMDs in Chimpanzee Lineage

To examine the possible effects of the removal of ancestral genomic sequences during the 663 chimpanzee lineage-specific ARMD events, we retrieved the pre-recombination sequences (i.e., unaltered orthologs) from the human genome. About 46% (305 of 663) of the ARMD events were located within known or predicted RefSeq genes (http://www.ncbi.nlm.nih.gov/mapview/map_search.cgi?taxid=9606), and five ARMD events generated 13 exonic deletions in six genes annotated as either demonstrably or putatively functional in the human genome. Among them, two ARMD events deleted exons from demonstrably functional genes in the *NBR2* (neighbor for *BRCA1* [breast cancer 1] gene 2) and *HTR3D* (5-hydroxytryptamine [serotonin] receptor 3 family member D) genes. While no alternative pre-mRNA spliced forms exist for the *NBR2* gene, the *HTR3D* gene shows three alternative pre-mRNA spliced forms in the human according to the ECR Browser (http://ecrbrowser.dcode.org). Among them, one of the *HTR3D* isoforms does not contain exon 3, which was deleted from the chimpanzee genome. Thus, chimpanzees could produce a similar protein to the *HTR3D* isoform mentioned above, because the ARMD event deleted the entire exon 3 and portions of some introns in the chimpanzee genome. However, we cannot rule out that the ARMD event has produced cryptic splicing sites causing either nonfunctionalization or subfunctionalization of HTR3D. The remaining three chimpanzee ARMD events generated exonic deletions in four putative human genes of unknown function *(LOC339766*, *LOC127295, LOC729351,* and *LOC645203).*

To further analyze the genomic sequences lost due to the ARMD process in the chimpanzee genome, we used the National Center for Biotechnology Information's (NCBI) UniGene utility (http://www.ncbi.nlm.nih.gov/sites/entrez?db=unigene) to look at the orthologous loci in the human genome, which contained sequences that would have been present in the chimpanzee genome if the ARMD events had not occurred. UniGene indicated that 164 ARMD events had caused deletions of coding sequence on the basis of expressed sequence tags (ESTs), although this number decreased to 94 when a high threshold indicating protein similarities (≥98% ProtEST) was selected (Table S1). This number is much higher than the exonic deletions in six genes generated by ARMD events reported above when RefSeq annotation was used instead.

### Structural Features of ARMD Events

**Figure 2:**
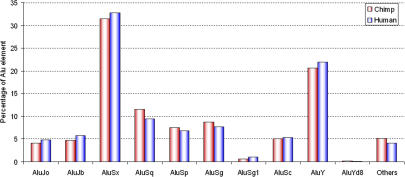
*Alu* Subfamily Composition in ARMD Events Proportion of all *Alu* elements involved in chimpanzee- and human-specific ARMD events (red and blue bars, respectively) that belong to each *Alu* subfamily as noted.

**Figure 3:**
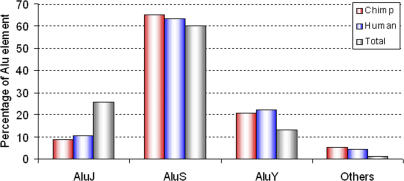
Comparison of *Alu* Subfamilies Involved in ARMD Events Proportion of *Alu* elements involved in chimpanzee-specific (red bars) and human-specific (blue bars) ARMD events versus proportion of total *Alu* elements in each subfamily in the chimpanzee genome (gray bars).

Ten different *Alu* subfamilies are associated with chimpanzee-specific ARMD events: *Alu*Jo, *Alu*Jb, *Alu*Sx, *Alu*Sq, *Alu*Sp, *Alu*Sg, *Alu*Sg1, *Alu*Sc, *Alu*Y, and *Alu*Yd8. Their composition and ratio in chimpanzee-specific ARMD events are remarkably similar to those in human-specific ARMD events (Figure 2). The *Alu* subfamily analysis shows that the number of elements from each *Alu* subfamily involved in the ARMD process is proportional to the genome-wide copy number of each *Alu* subfamily in the chimpanzee genome. For example, the *Alu*S subfamily has contributed the most to chimpanzee-specific ARMD events because it is the most successful *Alu* subfamily in the primate genome in terms of copy number. However, we found one exception to this rule; the *Alu*J subfamily is more ubiquitous than the *Alu*Y subfamily in both the chimpanzee and human genomes (Figure 3), but more members of the *Alu*Y subfamily were found to be involved in the ARMD process. The major expansion of the *Alu*J subfamily in primate genomes occurred ∼60 Mya, whereas the *Alu*Y subfamily expanded only ∼24 Mya [14,19,20]. On the basis of these ages, the individual members of the *Alu*J subfamily have likely accumulated more point mutations than those of the *Alu*Y subfamily. As a result, *Alu*Y copies have more sequence identity among them than do the *Alu*J copies, which results in increased involvement in ARMD events. In addition, we investigated intra-*Alu* subfamily recombination-mediated deletions for both the *Alu*J and *Alu*Y subfamilies. Of the 103 events involving at least one *Alu*J element in the ARMD event, only 15 (14.6%) involved recombination between two *Alu*J elements. The *Alu*Y subfamily shows a higher rate of intra-subfamily recombination than the *Alu*J subfamily, with 219 loci in which at least one *Alu*Y element was involved in the recombination event, and 57 (26%) that were between two *Alu*Y elements. This suggests that the rate of recombination between *Alu*Y elements is 1.8 times higher than that between *Alu*J elements. Taken together, this suggests that, in addition to the copy number of each *Alu* subfamily, the level of sequence identity between the individual *Alu* elements in the genome is also an important variable influencing ARMD events.

From a mechanistic viewpoint, four different types of recombination may occur between two *Alu* elements. An *Alu* element consists of left and right monomers. In the first type, comprising about 88% (583 of 663) of the ARMD events in our study, the recombination occurred between the same monomers of the two *Alu* elements. A second type of recombination occurred between two *Alu* elements in which one had previously integrated into the middle of the other. Such insertions are commonly found in both the chimpanzee and human genomes because each *Alu* element bears two endonuclease cleavage sites (5′-TTTT/A-3′) between its two monomers. About 8% (51 of 663) of the ARMD events in the chimpanzee genome are products of this second type of recombination. The third type of recombination, seen in 25 of the 663 events (∼4%), involved recombination between the left and right monomers on two separate *Alu* elements. The last type occurred between oppositely oriented *Alu* elements. Instances of this type of ARMD are very rare, found only in four of the 663 cases (0.6%). This style of recombination is likely to be uncommon because the stretch of sequence identity between two *Alu* elements oriented in opposite directions to one another is too short to frequently generate unequal homologous recombination. Instead, these two *Alu* elements are more likely to cause *Alu* recombination-mediated inversions or A-to-I RNA editing through the posttranscriptional modification of RNA sequences [21].

### Analysis of the ARMD “Hotspots”

**Figure 4:**
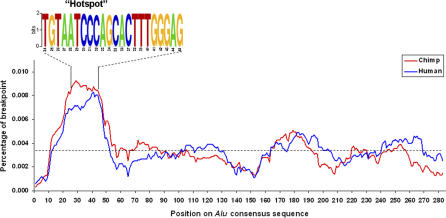
Recombination Breakpoints during Chimpanzee-Specific ARMD Events Percentage of ARMD events found to have breakpoints at different positions along an *Alu* consensus sequence. The “hotspot” region is represented by a conserved 22-bp nucleotide sequence found in 634 ARMD loci (the first and second types of ARMD events) using WebLogo analysis (http://weblogo.berkeley.edu). The dashed line represents the average percentage (0.0035%) of breakpoints across the entire length of the *Alu* consensus sequence.

To analyze the frequency of recombination at different positions along the length of the *Alu* elements (which we refer to as “recombination breakpoints”) at our ARMD loci, we aligned the two intact human *Alu* elements involved in each recombination event with the single chimeric *Alu* element from the chimpanzee genome (Figure S1). The windows between the two *Alu* elements range in size from 1 to 116 bp, with a mean of 20 bp and a mode of 22 bp. In general, the ARMD loci generated by intra-*Alu* subfamily recombination, as well as the recombination events between relatively young *Alu* elements, show longer stretches of sequence identity than others. Through this analysis, we identified a recombination “hotspot” on the *Alu* consensus sequence (5′-TGTAATCCCAGCACTTTGGGAGG-3′), located between positions 24 and 45 (Figure 4). This recombination hotspot is congruent with previous studies of gene rearrangements in the human LDL-receptor gene involving *Alu* elements [22], and with the pattern of recombination found in the 492 human-specific ARMD events [10]. Of these studies, the former suggested that the hotspot sequence (therein called the “core sequence”) might induce genetic recombination because it subsumes the prokaryotic *chi* sequence (the pentanucleotide motif CCAGC), which is known to stimulate *recBC*-dependent recombination [23]. We searched for and found the CCAGC motif at four places (positions 31–35, 85–89, 166–170, and 251–255) along the *Alu* consensus sequences. The percentages of breakpoints found at these positions are 0.00886%, 0.00336%, 0.00406%, and 0.00372%, respectively. Among these, the percentages of breakpoints found at the latter three positions are similar to the average percentage of breakpoints across the entire length of the *Alu* elements (0.0035%) in our ARMD events. The only spot where the motif is found that showed a substantially higher percentage of breakpoints is the one located at positions 31–35, which is within our proposed hotspot. Therefore, this motif may invoke, but does not seem to be essential for the generation of ARMD events.

Interestingly, the 22-bp hotspot sequence contains no CpG dinucleotides. These CpG dinucleotides have been shown to mutate approximately six times faster than other dinucleotides in *Alu* elements [24] due to cytosine methylation and subsequent deamination [25]. In addition, when we aligned the consensus sequences of the 10 different *Alu* subfamilies involved in ARMDs, we found that the hotspot sequence is located within the longest stretch of their conserved regions. Furthermore, using the software utility WebLogo [26], we confirmed that this 22-bp sequence is the most conserved region among *Alu* elements involved in ARMD events (Figure 4). Therefore, the recombination hotspot that we have identified, by virtue of having an increased level of conservation among the *Alu* subfamilies involved in the ARMDs in our study, has potentially allowed frequent recombination between *Alu* repeats from different *Alu* subfamilies to occur.

### Genomic Environment of ARMD Events

Most *Alu* elements located in the primate genomes that have been sequenced (e.g., human, chimpanzee, and rhesus macaque) exist in high-GC content regions [3–5], and also have high GC content (an average of ∼62.7%). Moreover, it has also been previously reported that human-specific ARMD events preferentially occur in areas of high GC content (∼45% GC content, on average) [10]. To analyze the genomic environment of chimpanzee-specific ARMD events, we estimated the GC content of 20 kb (±10 kb in either direction) of neighboring sequence for each ARMD locus. Our results indicate that the chimpanzee-specific ARMDs are similar to human-specific ARMDs in having a tendency to occur in GC rich regions (45.2% GC content, on average). This preference is correlated with the distribution of *Alu* elements involved in ARMDs (Figure 3) because the genomic distribution of ARMD events would in effect have an a priori dependence on the preferred locations of *Alu* elements after insertion of the different *Alu* subfamilies. About 74% of chimpanzee-specific ARMDs are associated with the older *Alu* subfamilies, *Alu*J and *Alu*S. Although young *Alu* subfamilies are found in AT-rich, gene-poor regions, the older *Alu* subfamilies are most often found in GC-rich, gene-rich regions [3]. This could account for the preferential occurrence of ARMD events in GC-rich regions. Moreover, the local rate of genomic recombination has been shown to be positively correlated with GC content [27], which may further explain the observed distribution of ARMD events. About 44% of genomic DNA deleted through ARMD events were *Alu* sequences in the human ortholog. This could indicate that regions of high local *Alu* element density within chromosomes are more likely to provide increased opportunities for local recombination, a trend previously noticed during analysis of the global genomic distribution of human lineage-specific ARMD events [10].

To further characterize the genomic environment of chimpanzee-specific ARMD events, we estimated the gene density of the genomic regions flanking each chimeric *Alu* element resulting from the process by extracting 4 Mb of flanking genomic sequences (±2 Mb in either direction), and counting the number of known or predicted chimpanzee RefSeq genes. The gene density of the flanking regions of chimpanzee-specific ARMD events is estimated to be, on average, one gene per 60.7 kb, which is similar to that of human-specific ARMD events (one gene per 66 kb). This indicates that the global distribution of chimpanzee-specific ARMD events is biased towards gene-rich regions, since the global average gene density in the chimpanzee genome is approximately one gene per 112 kb. To test for any relationship between the size of an ARMD and its flanking gene density or GC content, we performed a correlation test. While the *r*-values for both tests were negative, as would be expected given the danger of large deletions in gene-rich areas, the low *p*-values indicate that no significant correlation exists between the two variables in either test (gene density: *r* = −0.028; *p* = 0.472; GC content: *r* = −0.065; *p* = 0.095).

### Chimpanzee-Specific ARMD Polymorphism

In order to estimate the polymorphism rates in chimpanzees, we analyzed and amplified a total of 50 chimpanzee-specific ARMD loci on a panel composed of genomic DNA from 12 unrelated chimpanzee individuals (see Materials and Methods). Our results show that the polymorphism level of chimpanzee-specific ARMDs (28%) is about two times higher than the polymorphism rate of human-specific ARMD events (15%) [10], which is in general agreement with the polymorphism levels from previous studies of chimpanzee- or human-specific retrotransposons (e.g., *Alu* and L1 elements) [28,29].

### Incomplete Lineage Sorting and Parallel Independent ARMDs

**Figure 5:**
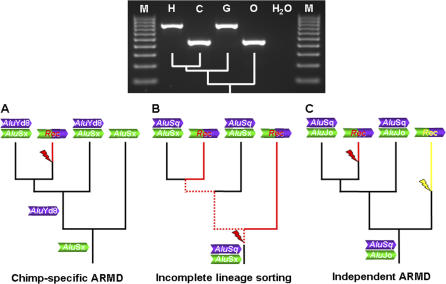
Incomplete Lineage Sorting and Parallel Independent ARMD Events The DNA template used in each reaction is listed on top of the gel chromatograph (M, 100-bp ladder; H, human; C, chimpanzee; G, gorilla; O, orangutan). The large and small sizes of PCR products indicate two *Alu* elements and one *Alu* element, respectively. The thunderbolts represent recombination events between two *Alu* elements, causing ARMDs. Possible scenarios that explain the observed chromatograph: (A) chimpanzee-specific ARMDs, (B) incomplete lineage sorting of an ARMD event, and (C) parallel independent ARMD events.

About 32% of the ARMD candidates were found to have ambiguous TSD structures and a triple alignment that proved too complex to assign ARMD status to the locus solely on the basis of our computational output. These loci were verified experimentally using PCR (see Materials and Methods) to determine the authenticity of the chimpanzee-specific ARMDs and identify false positives in the computational data, which were usually caused by human-specific *Alu* insertions. However, 16 ambiguous loci were identified at which human-specific *Alu* insertions were not present. In 11 of these loci, the human and gorilla genomes appear to have two *Alu* elements, while the chimpanzee and orangutan genomes have only one element at the orthologous position. DNA sequence analysis of the PCR products classified five of these 11 loci as chimpanzee-specific ARMDs, with the second of the two recombining *Alu* elements having integrated into the host genome after the divergence of orangutan and the common ancestor of humans, chimpanzees, and gorillas (Figure 5A). Four out of the 11 loci show a pattern consistent with incomplete lineage sorting, in which the ARMD event occurred before the divergence of great apes and was still polymorphic at the time of speciation. Subsequently, the chimeric *Alu* elements produced by these ARMD events became fixed in the chimpanzee and orangutan lineages while the two original *Alu* elements involved in the ARMDs were fixed in the human and gorilla genomes (Figure 5B). Incomplete lineage sorting has been reported in cases of retrotransposon insertion polymorphism involving closely related species [28,30]. In cases where the time between any genomic event and a subsequent speciation is very short, incomplete lineage sorting can easily occur. The remaining two of the 11 ambiguous loci were identified as parallel independent ARMD events in separate primate genomes by aligning the pre-recombination sequence and chimeric *Alu* elements (Figure 5C). These events suggest that orthologous loci may experience two independent lineage-specific ARMDs at different times (i.e., chimpanzee-specific ARMDs and orangutan-specific ARMDs).

In contrast, PCR analysis of the remaining five ambiguous loci (from the 16 referred to above) showed that humans and orangutans have two *Alu* elements, whereas chimpanzees and gorillas have only one at the orthologous position. Of these five loci, three showed a pattern suggesting incomplete lineage sorting events, while the other two were parallel independent ARMDs. For one of the loci displaying a parallel independent ARMD event, the structural characteristics of the two chimeric *Alu* elements resulting from independent recombination events are clearly different between the chimpanzee and gorilla genomes. The 574-bp chimpanzee genomic deletion occurred between the left monomer on the first *Alu* and the right monomer on the second *Alu*, whereas the 708-bp genomic deletion in the gorilla happened between the two left monomers of the two *Alu* elements.

These results indicate that at least ∼0.9% of chimpanzee-specific ARMD loci (2 of 233 loci which were analyzed by PCR) are shared by the gorilla genome and another ∼0.9% are shared by the orangutan genome, due to parallel independent ARMDs at two different time points in two separate primate genomes. As such, the presence of independently occurring ARMD events in both the human and chimpanzee genomes could lead to false negative events being missed during the previous analysis done by Sen et al. [10], although the frequency of such false negatives is likely to be very low. In addition, we believe that the human orthologs of the chimpanzee-specific ARMD loci represent sites predisposed for potential future ARMDs in the human genome that could generate human lineage-specific rearrangements and genetic disorders. Identifying putative ARMD hotspot genomic regions is not surprising based upon the frequency of *Alu*-mediated recombination events that have given rise to mutations in a number of different loci, including the *LDLR* and *MLL1* genes [11,31–33].

## Discussion

### Differential Level of Lineage-Specific ARMD Events

Despite the high level of overall similarity between their genomes, humans and chimpanzees have subtly different genomic landscapes because of alterations such as insertions, deletions, inversions, and duplications after their divergence from a common ancestral primate [8–11,34,35]. Although from a mechanistic viewpoint, the chimpanzee-specific ARMD events are similar to the human-specific ones, the total number and size of deletions are substantially different between the two lineages. One reason for the observed differences between these two lineage-specific ARMD patterns may be the increased genetic diversity of the chimpanzee population as compared to the human population, which is known to have experienced a significant reduction in its effective population size after the divergence of humans and chimpanzees [36], leading to a consequent reduction in genetic diversity. These results are supported by the higher polymorphism level for chimpanzee-specific ARMDs than human-specific ARMDs.

### Balance of Chimpanzee Genome Size

*Alu* elements as well as other retrotransposons can contribute to the size expansion of primate genomes by increasing their copy numbers and causing homology-mediated segmental duplications [37–39]. However, the retrotransposon-mediated increase in genome size is not unilateral, because several processes such as retrotransposon-mediated deletions and recombination-mediated deletions concurrently act in the opposite direction, causing reduction in genome size as well [8–10]. Retrotransposon-mediated negative control of genome size has been well documented in plants such as *Arabidopsis* and rice [40,41].

In this study, we analyzed the contribution of ARMDs to genome size regulation in the chimpanzee genome by estimating an *Alu*-mediated sequence turnover rate, which is the amount of sequence increase caused by chimpanzee-specific *Alu* insertions relative to the amount of reduction by the chimpanzee-specific ARMD process. The copy number of chimpanzee-specific *Alu* elements (i.e., those that inserted after the divergence of human and chimpanzee) is ∼2,340, accounting for ∼700 kb of inserted sequence in the chimpanzee lineage [3], while the amount of sequence deleted by chimpanzee-specific ARMDs is ∼771 kb. Therefore, within the past ∼6 million y, the genome size of chimpanzees has not expanded but rather has contracted by ∼71 kb, when considering the combined effects of *Alu* retrotransposition and recombination-mediated deletion (i.e., the *Alu*-mediated sequence turnover rate is more than 100% in the chimpanzee genome). This observation suggests that ARMD events efficiently counteract genomic expansion caused by novel *Alu* inserts in the chimpanzee genome when compared to the human genome. A previous analysis of human-specific ARMD events indicates that the *Alu*-mediated sequence turnover rate is ∼20% in the human genome [10]. This significantly different turnover rate between the two species could be explained by differences in the tempo of *Alu* amplification (i.e., higher *Alu* retrotransposition activity in the human genome) and rates of ARMD events (i.e., higher ARMD activity in the chimpanzee genome). Ultimately, it is worth noting that at least in the chimpanzee lineage, concurrent *Alu* insertion/ARMD mechanisms have balanced the gain and loss of sequences during *Alu-*mediated genomic alterations.

### Retrotransposition of Chimeric *Alu* Elements

To investigate whether chimeric *Alu* elements are able to retrotranspose in the chimpanzee genome, we tried to find progeny of the 663 chimpanzee-specific chimeric *Alu* elements using the BLAST-Like Alignment Tool (BLAT) program (http://genome.ucsc.edu/cgi-bin/hgBlat). However, we failed to recover any such elements in the chimpanzee genome for one or more of a number of reasons. First, *Alu* elements involved in ARMD events are expected to be relatively old (i.e., more than 6 million y) because our comparative analysis detects only ARMD events involving *Alu* elements that were inserted into the genome before the divergence of humans and chimpanzees. Therefore, most of the ARMD-associated *Alu* elements probably lost their ability to retrotranspose before the *Alu–Alu* recombination process. In reality, the contribution of chimpanzee-specific young *Alu* elements to the ARMD process may be extremely limited due to their low copy number (∼2,000 copies) in the chimpanzee genome [3]. Indeed, ARMD events generated by the relatively young *Alu*Y subfamilies account for 0.19% of the total *Alu*Y elements in the chimpanzee genome. Second, only a few source genes are responsible for new *Alu* subfamily amplification through retrotransposition. Although some *Alu* subfamilies (e.g., *Alu*Yc1) are still active in the chimpanzee genome [3,29], it is improbable that their source gene(s) are involved in the *Alu–Alu* recombination events. Similarly during an earlier analysis [10], we investigated the retrotransposition ability of 492 human-specific ARMD-generated chimeric *Alu* elements and were unable to recover their progeny as well.

### ARMD as an Endogenous Process Affecting Human and Chimpanzee Variation

Recently, the genomic relationship and genetic divergence between the human and chimpanzee genomes have been the subjects of extensive comparative genomic analyses on the basis of their respective draft genome sequences [3,35,42–44]. However, these studies have not focused on *Alu*-mediated genomic deletions in the chimpanzee lineage, aside from the 14 *Alu* retrotransposition-mediated deletions reported previously [9].

**Figure 6:**
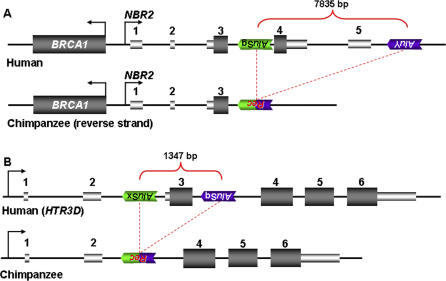
Exonic Deletions Caused by Two ARMD Events Black arrows represent the direction of transcription, and gray and black boxes indicate the noncoding exons and coding exons, respectively. Green and purple arrows indicate elements from two different *Alu* subfamilies, and dual-color arrows indicate chimeric *Alu*s generated by ARMD events (map is not drawn to scale). (A) An exonic deletion within the *NBR2* gene. The *Alu*Sg and *Alu*Y elements are located within the third intron and the 3′ flanking sequence, respectively, in the human genome. The exon4 sequence is deleted due to an ARMD event in the chimpanzee lineage. (B) An exonic deletion within the *HTR3D* gene. The *Alu*Sx and *Alu*Sq elements are located within the second and third introns, respectively, in the human genome. The exon3 sequence, which includes the initiation codon ATG, is deleted due to an ARMD event in the chimpanzee lineage.

Thus, our study forms the first comprehensive analysis of recombination-mediated genomic alteration by *Alu* elements in a nonhuman primate (chimpanzee) lineage. We found 305 chimpanzee-specific deletions within protein-coding genes as annotated by the RefSeq gene annotation database, 299 genes from which introns were deleted, and six genes in which thirteen exons were deleted. Remarkably, two chimpanzee-specific ARMD events deleted exons from genes demonstrably functional in the human lineage (*NBR2* and *HTR3D*), providing direct proof that the ARMD process contributes to creating phenotypic differences between humans and chimpanzees. The *NBR2* gene is located near the *BRCA1* gene on Chromosome 17, which is responsible for tumor repressor activity in the human genome, and shares a common promoter for transcription, forming a bidirectional transcriptional unit with *BRCA1.* Although the complete *NBR2* cDNA sequence is ∼1.3 kb, it has a short open reading frame (112 amino acids), and is subject to nonsense-mediated decay [45,46]. In humans, this gene is suppressed by a non–tissue-specific protein complex that binds to its first intron (i.e., the 18-bp repressor element) [47]. However, in the chimpanzee lineage, an ARMD event occurred between the third intron and the 3′ flanking region, causing an exonic deletion (Figure 6A). Thus, this ARMD event could potentially inhibit *NBR2* gene expression in the chimpanzee genome, regardless of whether or not the repressor element is present. Although the exonic deletion of the *NBR2* gene has been independently reported through a comparative analysis of cancer genes between the human and chimpanzee genomes, the previous analysis did not report what caused this genetic difference between human and chimpanzee genomes [48]. Our study of chimpanzee-specific ARMDs illuminates the underlying molecular mechanism for this deletion.

A chimpanzee-specific ARMD event also deleted the first coding exon of *HTR3D,* a functional gene in humans (Figure 6B). This gene belongs to the 5-HT_3_ serotonin receptor-like gene family, which has been recently characterized [49]. The 5-HT_3D_ subunit is not a functional receptor on its own (i.e., a homomeric receptor), but when it binds to the 5-HT_3A_ subunit to form the heteroligomeric receptor, 5-HT, maximum response is significantly increased as compared to the homomeric 5-HT_3A_ receptor [50]. *HTR3D* is primarily expressed in the gastrointestinal tract [50], where serotonin is synthesized extensively [51]. We speculate that the exonic deletion in this gene caused by the chimpanzee-specific ARMD event may lead to a reduction in serotonin levels in the chimpanzee lineage, and thus have an impact on physiological variation between the human and chimpanzee lineages.

The analyses using the RefSeq and UniGene annotations (see Results) indicate that ARMD events could have affected the expression of many genes. Moreover, intronic or intergenic deletions caused by ARMD events may also affect the levels of gene expression in both the human and chimpanzee genomes through alteration of splicing patterns and loss of transcription factor binding sites, further contributing to the divergence of the human and chimpanzee lineages. Additional studies of the functional genomics of the genes altered in both human and chimpanzee ARMD events will be instructive and provide new insight into the genetic and phenotypic differences between the two species.

### Conclusion

Retrotransposon-mediated genomic rearrangement could be one of the major factors responsible for the lineage-specific changes in genomes that ultimately lead to speciation. Comparative investigations of the ARMD events apparent between the human and chimpanzee genomes indicate that this process plays an important role in the biological differences between humans and chimpanzees, and provides a reliable record of lineage-specific evolutionary histories due to the nearly homoplasy-free nature of these mutations. Moreover, in the chimpanzee lineage, the chimpanzee-specific ARMD process has completely counteracted the genomic expansion caused by new *Alu* inserts since the divergence of the chimpanzee and human lineages. The existence of parallel independent ARMD events found at the orthologous loci of some of the 663 chimpanzee-specific ARMD events suggest that other chimpanzee-specific ARMD orthologs in humans may be predisposed to undergo recombination between the two *Alu* elements in the future. These ARMD orthologous loci may be sites of unstable structure in humans as well as other apes, because they still preserve the pre-recombination structure that has proven itself susceptible to unequal recombination in the chimpanzee lineage.

## Materials and Methods

### Computational search and manual inspection of chimpanzee-specific ARMD loci.

To computationally screen the chimpanzee genome for potential ARMD loci, we used a technique previously described by Sen et al. [10] in a study of human lineage-specific ARMD events, with the distinction that, for this analysis, the query and target genomes were reversed. In summary, we extracted 400 bp of 5′ and 3′ flanking sequence for all chimpanzee *Alu* elements (PanTro1; November 2003 freeze) and joined the two 400 bp sequences to form a single “query” sequence. A best match for each query sequence was determined by using BLAT [52] against the reference human genome (hg17; May 2004 freeze). Then, the sequence in the human genome (the “hit”) found between the orthologs of the two 400 bp stretches of the query was extracted and aligned with the chimpanzee *Alu* element sequence initially used to design the query (the “query *Alu*”) using a local installation of the NCBI bl2seq utility.

One hallmark of de novo *Alu* insertion is the presence of TSDs flanking each side of the *Alu* element, generated by the target-site primed reverse transcription process [1,53–55]. However, the single chimeric *Alu* element created by an ARMD event lacks matching TSD structures in the chimpanzee because it is comprised of fragments from a pair of *Alu* elements with mutually unique TSDs at the orthologous ancestral locus [10]. If a potential ARMD locus exhibited the structures of a valid ARMD as described by Sen et al. [10], we accepted the computational detection as an authentic ARMD locus. In addition, we used the BLAT software utility [52] to compare the human, chimpanzee, and rhesus macaque genomes at each potential ARMD locus. If the two *Alu* elements in the human genome that are considered to be the pre-recombination *Alu* elements for an ARMD locus are shared with the rhesus macaque genome at orthologous loci, despite the presence or absence of TSDs, the single *Alu* element remaining at the orthologous chimpanzee locus is most likely a chimeric element generated an ARMD event. On the basis of these features, we manually inspected 1,538 potential ARMD loci retrieved by the computational data analysis. However, some loci displayed ambiguous TSD structure or remained ambiguous after analysis using the triple alignment. These loci were subjected to PCR analysis and, if necessary, DNA sequencing in order to confirm or eliminate each as being products of bona fide ARMD events.

### PCR amplification and DNA sequence analysis.

PCR analysis was performed using four different primate species as templates. The cell lines used to isolate DNA samples corresponding the primate species are as follows: human *(Homo sapiens)* HeLa (CCL2; American Type Culture Collection [ATCC], http://atcc.org), common chimpanzee “Clint” (*Pan troglodytes;* NS06006B), gorilla (*Gorilla gorilla;* AG05251) and orangutan (*Pongo pygmaeus;* AG05252A). To evaluate polymorphism rates, we amplified 50 randomly selected ARMD loci on a common chimpanzee population panel composed of 12 unrelated individuals of unknown geographic origin obtained from the Southwest Foundation for Biomedical Research (San Antonio, Texas, United States).

Oligonucleotide primers for the PCR amplification of ARMD events were designed using the Primer3 utility (http://www-genome.wi.mit.edu/cgi-bin/primer/primer3_www.cgi). The sequences of the oligonucleotide primers, annealing temperatures, and PCR product sizes are shown in Table S2. Each PCR amplification was performed in 25-μl reactions using 10–50 ng DNA, 200 nM of each oligonucleotide primer, 200 μM dNTPs in 50 mM KCl, 1.5 mM MgCl_2_, 10 mM Tris-HCl (pH 8.4), and 2.5 U *Taq* DNA polymerase. Each sample was subjected to an initial denaturation step of 5 min at 95 °C, followed by 35 cycles of PCR at 1 min of denaturation at 95 °C, 1 min at the annealing temperature, and 1 min of extension at 72 °C, followed by a final extension step of 10 min at 72 °C. PCR amplicons were loaded on 1%–2% agarose gels, depending on the amplicon sizes, stained with ethidium bromide, and visualized using UV fluorescence. In cases where the expected size of the PCR product was greater than 1.5 kb, i*Taq* (Bio-Rad, http://www.bio-rad.com) or *Ex Taq* polymerase (TaKaRa, http://www.takara-bio.com) were used, following the manufacturer's suggested protocols.

When necessary, individual PCR amplicons were gel purified using the Wizard gel purification kit (Promega, http://www.promega.com) and cloned into vectors using the TOPO-TA Cloning kit (Invitrogen, http://www.invitrogen.com) according to the manufacturer's instructions. DNA sequencing was performed using dideoxy chain-termination sequencing [56] on an Applied Biosystems ABI3130*XL* automated DNA sequencer (Applied Biosystems, http://www.appliedbiosystems.com). Raw sequence reads were assembled using DNASTAR's Seqman program in the Lasergene version 5.0 software package (http://www.dnastar.com).

### Analysis of flanking sequences.

For each chimpanzee-specific ARMD locus, 10 kb of flanking sequence upstream and downstream were collected using a combination of in-house Perl scripts and the nibFrag utility bundled with the BLAT software package. The GC content of the flanking regions of each ARMD locus was calculated by analyzing the combined 20 kb of flanking sequence using another in-house Perl script, which excluded Ns from the analysis. Gene density around individual ARMD loci was estimated using the NCBI Map Viewer utility, run on Build 2.1 of the Pan troglodytes genome (http://www.ncbi.nlm.nih.gov/mapview/map_search.cgi?taxid=9598). The neighboring 2 Mb of sequence 5′ and 3′ to each chimeric chimpanzee *Alu* element was analyzed, and the number of genes found within this combined 4 Mb were noted. All computer programs used are available from the authors upon request.

## Supporting Information

Dataset S1 Dataset of 663 ARMD Loci (2.2 MB TXT)

Figure S1 Sequence Alignment of a Chimeric Chimpanzee *Alu* and Two Intact Human *Alu* Elements The chimeric chimpanzee *Alu* sequence is shown at the top. The sequences of the intact human *Alu*Sx and *Alu*Jb involved in the ARMD events are shown below. The dots below represent the same nucleotides as the chimeric chimpanzee *Alu* sequence, and the dashes represent the gaps. A yellow box on the sequences denotes the recombination window.
(49 KB DOC)

Table S1 Exonic Deletions Caused by ARMD Events Based on the UniGene Utility (41 KB XLS)

Table S2 Oligonucleotide Primer Information for Chimpanzee-Specific ARMDs (69 KB XLS)

### Accession Numbers

The gorilla and orangutan DNA sequences generated during the course of this study have been deposited in GenBank (http://www.ncbi.nlm.nih.gov/Genbank) under accession numbers EF682150–EF682182. The GenBank accession numbers for the three *HTR3D* isforms discussed in this article are NM_182537, BC101090, and AJ437318.

## Abbreviations

ARMD: *Alu* recombination-mediated deletion
Mya: million y ago
TSD: target site duplication
