# Supplementary material for: Alu Recombination-Mediated Structural Deletions in the Chimpanzee Genome
Source: PLoS Genet. 2007 Oct 19;3(10):e184. doi: 10.1371/journal.pgen.0030184 (PMC2041999; doi:10.1371/journal.pgen.0030184)
Supplement: Supplementary file 1 [file pgen.0030184.sg001.doc]

                         10        20        30        40        50        60        70        80               
                ....|....|....|....|....|....|....|....|....|....|....|....|....|....|....|....|
Chimeric Alu    GGCTGGGTGCAGTGGCTCACGCCTGTAATCCCAGCACTCTGGGAGGCTGAGGTGGGTG-ATCACTTGAGCTGAGGAGTTT 
AluSx (First)   ......................................T......A.CA...C.....G..........G.C.......C 
AluJb (Second)  -......CATGA.....TCTA.....................................-..................... 

                         90       100       110       120       130       140       150       160        
                ....|....|....|....|....|....|....|....|....|....|....|....|....|....|....|....|
Chimeric Alu    GAGACCAGCCTGGGCAATATAGAGAAACCCCACCTCTACAAAAAATACAAAAATTACCCAGGCATGGCAATGTGTGCCTC 
AluSx (First)   .............C...C..G.T........GT...C..T................G..G..TGCA.TGGCACACA...A 
AluJb (Second)  ................................................................................ 

                        170       180       190       200       210       220       230       240       
                ....|....|....|....|....|....|....|....|....|....|....|....|....|....|....|....|
Chimeric Alu    TAGTCCCAGCTACTCAGGAGGCTGAGGTGGGAGGATCACTTGAGCCTGGGAGGTTTAGGCTGCAGTGAGCCATGATCGTG 
AluSx (First)   ...............G...........CA....A.........A..C......------T............A..CT... 
AluJb (Second)  ................................................................................ 

                        250       260       270       280       290            
                ....|....|....|....|....|....|....|....|....|....|....|...
Chimeric Alu    CCACTGCACTCCAGCCTGGGCGAGAGAATGAGACCCTGTCTCAAAATAAATAAATAAA 
AluSx (First)   .......................C...GCA....T.CA......-------------- 
AluJb (Second)  .......................................................... 


Figure S1. Sequence alignment of a chimeric chimpanzee Alu and two intact human Alu elements.
